# Ocular Ultrasound as an Easy Applicable Tool for Detection of Terson's Syndrome after Aneurysmal Subarachnoid Hemorrhage

**DOI:** 10.1371/journal.pone.0114907

**Published:** 2014-12-11

**Authors:** Patrick Czorlich, Till Burkhardt, Volker Knospe, Gisbert Richard, Eik Vettorazzi, Lars Wagenfeld, Manfred Westphal, Jan Regelsberger, Christos Skevas

**Affiliations:** 1 Department of Neurosurgery, University Medical Center Hamburg-Eppendorf, Hamburg, Germany; 2 Department of Ophthalmology, University Medical Center Hamburg-Eppendorf, Hamburg, Germany; 3 Department of Medical Biometry and Epidemiology, University Medical Center Hamburg-Eppendorf, Hamburg, Germany; Massachusetts Eye & Ear Infirmary, Harvard Medical School, United States of America

## Abstract

**Introduction:**

Intraocular hemorrhage in patients suffering from aneurysmal subarachnoid hemorrhage is known as Terson's syndrome and is an underestimated but common pathology. We therefore designed a prospective single-blinded study to evaluate the validity of ocular ultrasound compared to the gold standard indirect funduscopy in the diagnosis of Terson's syndrome.

**Material and Methods:**

Fifty-two patients (104 eyes in total) suffering from aneurysmal subarachnoid hemorrhage were enrolled in this study. Two investigators independently performed a single-blinded ocular ultrasound using a standard intensive care ultrasound system to detect an intraocular hemorrhage. Indirect funduscopy following iatrogenic mydriasis served as the gold standard for confirmation or exclusion of an intraocular hemorrhage. Statistical analyses were performed to evaluate the sensitivity and specificity, positive and negative predictive values of the method as well as the learning curve of ocular ultrasound.

**Results:**

Indirect funduscopy detected Terson's syndrome in 11 of 52 (21.2%) respectively in 21 of 104 (20.2%) eyes in patients suffering from subarachnoid hemorrhage. Sensitivity and specificity increased with the number of ocular ultrasound examinations for both investigators, reaching 81.8% and 100% respectively. Positive and negative predictive values were different for both investigators (63.6% vs. 100% positive and 100% vs. 95.7% negative) but were both correlated to the amount of intraocular hemorrhage. A low Glasgow Coma scale (p = 0.015) and high Hunt & Hess grade (p = 0.003) was associated with a higher rate of Terson's syndrome.

**Conclusions:**

Ocular ultrasound using standard ultrasound equipment has been confirmed as a reliable, easy-to-handle bedside screening tool for detecting Terson's syndrome. Nevertheless funduscopy remains the gold standard to detect Terson's syndrome.

## Introduction

Subarachnoid hemorrhage (SAH) is most likely caused by the rupture of an intracranial aneurysm and may be followed by an intraocular hemorrhage (IOH) [1]. IOH following SAH was first described by Moritz Litten [2] in 1881 and was later named after French ophthalmologist Albert Terson [3]. The rate of Terson's syndrome (TS) may be as low as 12% but has been reported to hit the 50% level, thus being suspected to be far underestimated. TS appears to correlate with the extent of SAH for some unknown reason [4]–[6] and may lead to long-term visual impairment accompanied by proliferative retinopathy, retinal breaks, retinal detachment and cataracts [7]. Although patients achieve an excellent clinical outcome with medical management alone, vitreoretinal surgery is necessary in some of these patients to improve Quality of Life. [8]–[10]. Therefore, correct diagnosis of TS is of significant prognostic value in patients suffering from SAH for the ophthalmological clinical course and neurological rehabilitation [5]–[7]. TS is commonly diagnosed by indirect funduscopy, which requires iatrogenic mydriasis [5], [11]. However, as unilateral and bilateral pupillary dilation is also a sign of sudden increase of intracranial pressure (ICP) [12]–[13] requiring urgent diagnostic and therapeutic considerations, funduscopy is not a feasible method for critically ill SAH patients. In this context ocular ultrasound presents as a well-known but less utilized diagnostic tool available for all clinics without specialized ophthalmological service [7], [14], [15]. We therefore designed a prospective single-blinded study to evaluate the validity of ocular ultrasound compared to indirect funduscopy in the diagnosis of TS. Aim of this study was to scrutinize if ocular ultrasound may be established as a standard diagnostic tool in non-specialized intensive care and neurological rehabilitation units to screen for ocular bleedings and thus preventing worse clinical outcome with visual impairment.

## Materials and Methods

All patients admitted with the diagnosis of SAH in a 14 month period were classified as potential candidates for this study. The diagnosis of SAH was confirmed by cranial CT imaging and/or magnetic resonance imaging and/or lumbar puncture. Site and morphology of the aneurysm was specified by four vessel cerebral digital subtraction angiography. Patients were not included if an aneurysmal bleeding could be excluded and if SAH was judged to be caused by other non-aneurysmal pathologies. Severity of SAH was categorized according to the initial Glasgow coma scale (GCS), Hunt & Hess grade (H&H) as well as the Fisher Scale. Further analyses were performed on patient sex and age, and the localization, diameter and quantity of aneurysms.

**Figure 1:**
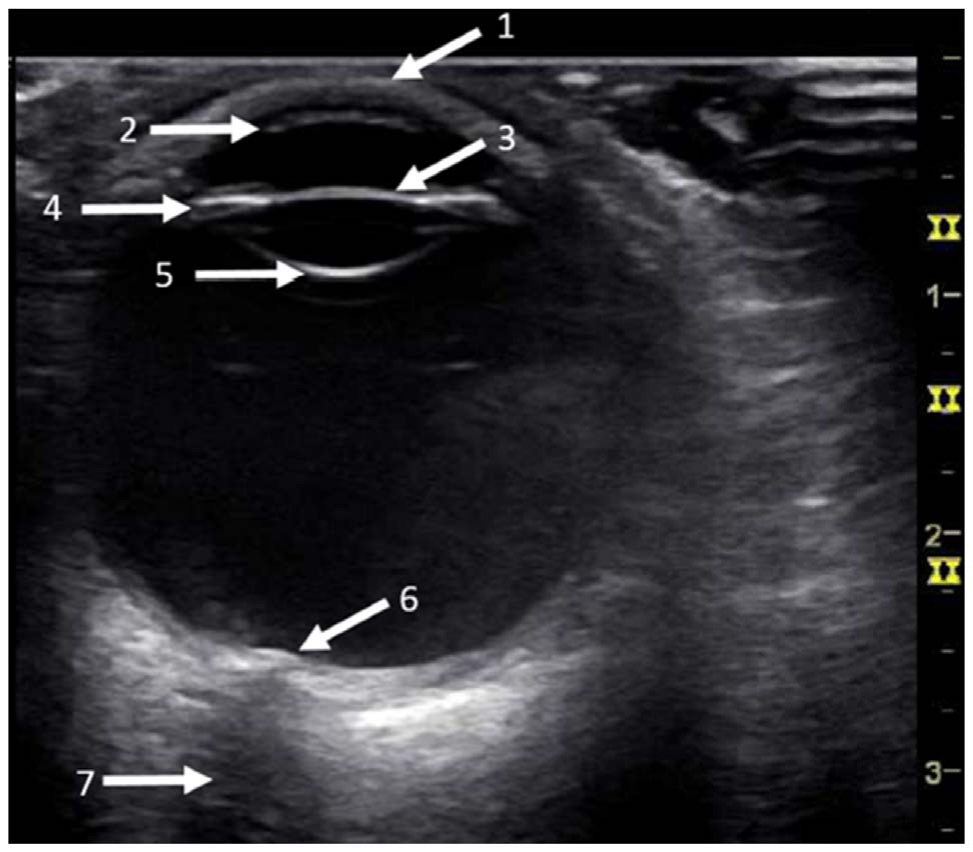
Ocular Ultrasound of a normal eye. The ultrasound probe is applied to the closed eye and transmission gel used to avoid any pressure on the eye. The normal eye (here right eye) appears as a round hypoechoic (grey-black) structure, and the cornea (2) is a thin hyperechoic (white) layer next to the eyelid (1). This is adjacent to the anterior chamber (hypoechoic water-filled cavity). Below the chamber, a hyperechoic iris and ciliary bodies (4) follow with the anterior (hyperechogenic) reflection of the lens (3). The lens itself is hypoechogenic with a smaller reflection on the rear side (5). The vitreous body is hypoechogenic due to its water-filled cavity. Retina may not be differentiated from choroidal layers, while the optic nerve appears as a hypoechoic linear structure (7) entering the vitreous chamber (6).

A standard ultrasound system (General Electrics, Vivid S6; GE 8L-RS, General Electrics Healthcare, Chalfont St Giles, United Kingdom) with high-resolution linear probe (10 MHz) was used to simulate an examination in regular care hospitals outside high specialized medical centers. Standard water-soluble ultrasound transmission gel was applied to the closed eyelid of the patient so that the transducer did not touch the eyelid avoiding any pressure to the ocular bulb. (Fig. 1)

All participating patients were examined by two independent investigators, a neurosurgical resident with no special expertise in ocular ultrasound (OUS) and a consultant neuro-ophthalmologist. The neurosurgical resident was introduced to OUS by the consultant neuro-ophthalmologist in a hands-on-instruction training performing 20 OUS in normal healthy individuals. The consultant neuro-ophthalmologists performed more than 1000 OUS prior to the start of the study. OUS results were recorded in a standardized data sheet, blinded and followed by indirect funduscopy serving as reference and which was performed by a consultant neuro-ophthalmologist (iatrogenous mydriasis with Tropicamid, Mydriaticum Stulln, Pharma Stulln GmbH, Stulln, Germany). Any kind of IOH, including sub-retinal, intra-retinal, pre-retinal (summarized as retinal hemorrhages), sub-hyaloid, or vitreous hemorrhages were classified as TS. A dense vitreous hemorrhage was defined as severe IOH. Investigators were informed about the correct result of their OUS after OE, with the expectation that this would accelerate the learning curve.

### Ethics statement

The study was approved by the local ethic committee at the medical council of the state of Hamburg (Ethik-Kommission der Ärztekammer Hamburg - PV4079). Written informed consent was obtained by either the patient or the legal representative prior to OUS and ophthalmoscopy examination.

### Definitions of Measurement

Primary endpoint of this study was description of sensitivity, specificity, positive and negative predictive value and accuracy of ocular ultrasound in the detection of Terson syndrome. The secondary endpoint was descriptionof the learning curve for the detection of Terson's syndrome by ocular ultrasound.

### Statistical Analysis

Depending on the scale of measurements, Student's *t*-test or chi-squared tests (ordered factors were tested using a trend test) were used for statistical analysis to examine correlations between the parameters. Sensitivity, specificity, positive and negative predictive values as well as accuracy had been calculated from appropriate cross-tabulations. The hypothesized effect of a learning curve with increasing experience was estimated by a Mantel-Haenszel test after dividing the cohort into two blocks (patients 1–26 and 27–52). The level of statistical significance was set at *p*<0.05. All analyzes were performed using IBM SPSS Statistics 19, Chicago, IL, USA.

## Results

### Rate and Risk factors of Terson's syndrome

Of a total of 92 SAH patients, 52 patients (34 women and 18 men) with a mean age of 54.1 years±13.0 years (range 23–81) were enrolled in this study. Mean age in patients suffering from TS was 50.5 years±9.6 years (range 34–75) and 55.0 years±13.7 years (range 23–81) in patients without TS (*p* = 0.09). The incidence of TS in our study group was 21.2% (11 out of 52 patients). In total, 21 eyes were affected by an IOH, 9 eyes by dense vitreous hemorrhage and 12 eyes by retinal hemorrhages. Dense vitreous hemorrhage was seen in both eyes of four patients. One patient suffered from dense vitreous hemorrhage in one eye and retinal hemorrhage in the other eye. The incidence of TS increased with the severity of SAH. Lower initial GCS (*p* = 0.015) and higher Hunt & Hess grade (*p* = 0.003) are associated with a higher rate of TS. Sex and Fisher grade had no significant impact on the rate of TS (Table 1). The diameter of the aneurysm (*p* = 0.429), number of aneurysms (*p* = 0.795) and the location of the aneurysm (*p* = 0.116) had no significant impact on the rate of TS.

**Table 1:** Distribution of sex, initial Glasgow Coma scale (GCS), Fisher grade in initial cranial CT imaging scan and Hunt & Hess grade (based on reference 4).

Characteristic		No. of patients		*p*-value
		Without Terson's syndrome	With Terson's syndrome	
Sex	Male	14 (77.8%)	4 (22.2%)	0.892
	Female	27 (79.4%)	7 (20.6%)	
Initial GCS	≥8	31 (88.6%)	4 (11.4%)	0.015
	<8	10 (58.8%)	7 (41.2%)	
Fisher grade initial cCT	1	2 (100%)	0 (0%)	0.180
	2	1 (100%)	0 (0%)	
	3	13 (86.7%)	2 (13.3%)	
	4	25 (73.5%)	9 (26.5%)	
Hunt & Hess grade	I	13 (100%)	0 (0%)	0.003
	II	8 (100%)	0 (0%)	
	III	8 (72.7%)	3 (27.3%)	
	IV	9 (60.0%)	6 (40.0%)	
	V	3 (60.0%)	2 (40.0%)	

### Analysis of sensitivity, specificity, positive and negative predictive value and accuracy

**Figure 2:**
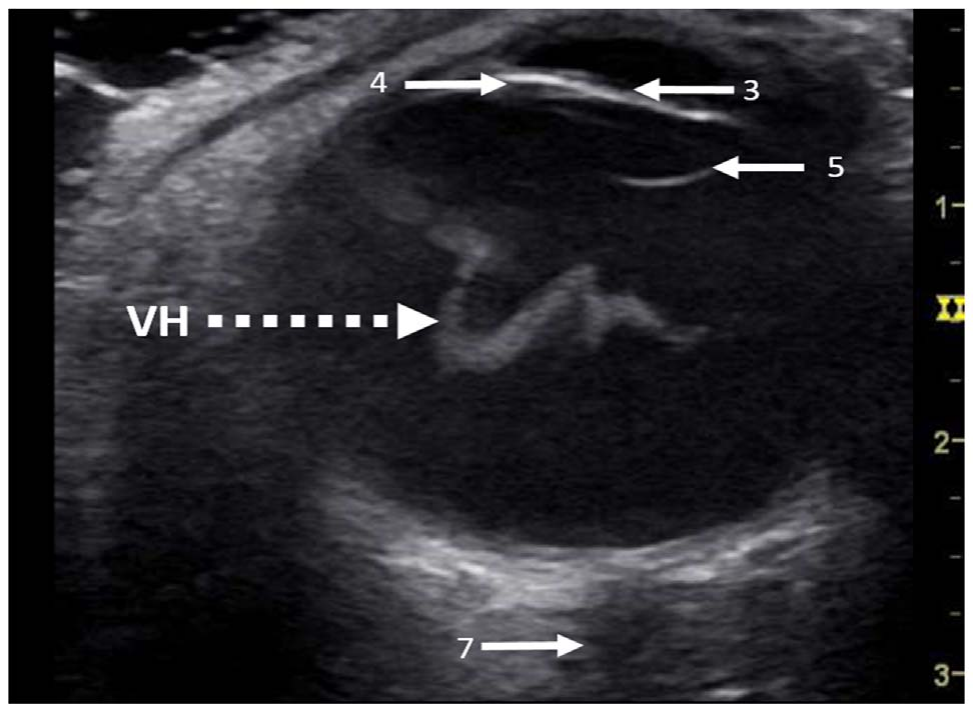
Ocular ultrasound examination of a vitreous haemorrhage (VH) in the left eye. In this case Terson's syndrome appeared as a hyperechogenic membrane caused by clotted blood within the vitreous body. According to Fig. 1 iris and ciliary bodies (4) as well as the anterior (3) and posterior reflection (5) of the lens and the optic nerve (7) are visible.

Investigator I detected 11 out of 21 IOH (7 vitreous and 4 retinal hemorrhages) with OUS (sensitivity 52.4%, positive predictive value 57.9%), while 75 out of 83 eyes without IOH were stated to have no TS (specificity 90.4%, negative predictive value 88.2%, overall accuracy 82.7%; Fig. 2). In 8 eyes, an IOH was diagnosed by OUS without the existence of TS (false positive) and in 10 eyes a TS was not detected by OUS (false negative). Investigator II was found to have a specificity of 100% (83 out of 83 eyes) with a negative predictive value of 84.7% and a sensitivity of 21.1% (4 out of 19 IOH; 3 vitreous and 1 retinal hemorrhages) with a positive predictive value of 100% (an accuracy of 85.3%).

Specificity was 89.2%, and negative predictive value of OUS in dense vitreous hemorrhage was 97.6% for investigator I (with an accuracy of 88.5%). For investigator II, the rate was slightly higher, with a specificity of 100% and negative predictive value of 94.9% (with an accuracy of 95.1%). Sensitivity and positive predictive value increased in both investigators (investigator I; sensitivity 81.8%, positive predictive value 47.4%, investigator II; sensitivity 44.4%, positive predictive value 100%).

### Analysis of Learning Curve

The learning curve (patients 1–26 vs. patients 27–52) of investigator I increased significant (*p*<0.001) and also learning curve of investigator II improved (no reliable calculation due to sample size). Details of learning curve are presented in Table 2.

**Table 2:** Learning curve of sensitivity, specificity, positive and negative predictive value and accuracy of investigator I and II.

Characteristics	Investigator I		Investigator II	
	Pts. 1–26	Pts. 27–52	Pts. 1–26	Pts. 27–52
Sensitivity	28.6%	100%	7.1%	60.0%
Specificity	89.5%	91.1%	100%	100%
Positive predictive value	50.0%	63.6%	100%	100%
Negative predictive value	77.3%	100%	74.5%	95.7%
Accuracy	73.1%	92.3%	75.0%	96.0%

Pts.  =  patients.

## Discussion

Terson's syndrome is common in patients suffering from SAH, and its rate is reported to reach 46% [4]–[6]. It is associated with a low initial GCS and a high Hunt & Hess grade and tends to affect women more than men [4]–[5], [16]. TS may lead to proliferative retinopathy, retinal breaks, retinal detachment and cataracts leading to persisting visual impairment [7]. More severe forms of TS with dense vitreous hemorrhage require ophthalmologic intervention by pars plana vitrectomy [4]. Today, indirect funduscopy is the gold standard for diagnosing TS, but iatrogenic mydriasis may mask life threatening complications, especially in neurocritically ill patients [12]. The intention of our prospective study was to evaluate the sensitivity and specificity of ocular ultrasound in the daily routine detecting TS to encourage non specialized intensive care and neurological rehabilitation units to screen for TS.

Our results have shown that OUS as a bedside, non-invasive tool is of high diagnostic value and provides high accuracy detecting ocular pathologies, confirming previous findings by Blaivas et al. and others [14], [15], [17], [18]. This is of special interest to neuro-intensive care and neurological rehabilitation units, as OUS is not routinely performed in SAH patients, where IOH followed by temporary or permanent visual acuity may affect nearly half of all patients [6]. Our study has confirmed that OUS is a safe and useful technique in diagnosing pathological changes of the ocular globe. Its ease of handling is accompanied by a fast accelerating learning curve, further improving diagnostic reliability.

Regarding the mechanical and biological risks of exposing the eye to high frequency probes, sonography side effects have not been reported up to now. In animal studies, rabbit corneas and retinas were exposed to ultrasound ranging from 10 to 60 MHz for up to 30 minutes with no deleterious effects [19]. Nevertheless, mechanical energy is transmitted into the eye and more than 4–10×10^6^ oscillations may warm the intraocular fluid. Therefore it is important to limit the examination time and gain according to the ALARA (‘as low as reasonably applicable’) principle. Compared to ultrasonography in pregnancy, which appears to be safe regarding the bio effects on fetal growth and development, the eye is a far less motile object but investigated here by higher frequencies (10–20 MHz) in OUS [20]–[21]. To summarize the scarce data available from animal studies and human observations careful handling of the setting is required. Globe rupture is considered to be a clear contraindication for OUS due to the risk of extrusion of ocular contents [22]–[24].

Blaivas et al. examined the accuracy of bedside ultrasound in the detection of ocular pathologies. Of the 61 patients enrolled in the study, 26 were found to have intraocular pathologies including retinal detachments, central retinal artery occlusion and lens dislocations. In this study a sensitivity of 100%, specificity of 97.2%, positive predictive value of 96.2% and a negative predictive value of 100% were observed [14]. In contrast to our study, the physicians here were not blinded and patients were admitted with ocular trauma or acute visual change most likely excluding minor pathological changes [14]. Parchand et al. retrospectively confirmed an overall sensitivity of 92.31% and a specificity of 98.31% in evaluating pre-surgical OUS in 130 patients including various ocular injuries, diabetic vitreous hemorrhage, endophthalmitis and other causes of vitreous hemorrhages [25]. The lower sensitivity and specificity observed in our series is explained by the blinded study design where both investigators were not aware of both, the SAH grade and visual acuity of the patient. Small intra-retinal, pre-retinal or sub-hyloidal bleedings may remain undetected. Other bleedings may appear as minimal thickening of retinal membranes misleading a correct diagnosis [26], while dense vitreous hemorrhage can be more easily identified.

Cranial computed tomography may also be used to detect TS in SAH patients, as these patients repeatedly undergo cranial CT imaging for other reasons [27]. A recent study of 121 patients diagnosing TS by cranial CT imaging was found to have a sensitivity of 42% and a specificity of 97%. These results are comparable to our findings but are associated with an unacceptably high radiation dose of the ocular bulb [16].

Our study has shown that with increasing experience in OUS diagnostic accuracy improves in a short time period. After 26 patients had been investigated, sensitivity (from 60% to 100%) and specificity (from 91.1% to 100%) increased significantly, confirming a rapid learning curve. This should encourage neuro-intensive care physicians to become familiar with OUS to diagnose IOH, thereby differentiating between a pathology requiring immediate ophthalmologic consultation (such as retinal detachment and intraocular foreign bodies) from those findings (such as vitreous hemorrhage or retinal hemorrhage) which can be followed up on an outpatient basis [14].

The incidence of TS may often be underestimated due to the severity of brain injury or intracranial hemorrhages associated with life-threatening conditions. In addition, full ophthalmological backup and rapid consultation is not always available during primary care of neuro-intensive care patients. Therefore only a minority of TS in SAH-patients will be diagnosed in an early stage and thus may shift this undetected diagnosis to rehabilitation units delaying full recovery. [7]. Even timing of ophthalmological interventions in TS remains unclear [28] and spontaneous clearance of vitreous hemorrhage can be expected within 10–12 months, vitreoretinal surgery is necessary in some of these patients to prevent epiretinal membrane formation, macular holes, proliferative vitreoretinopathy and retinal detachment [4]. In this context, ocular ultrasound is a very important tool to augment the diagnostic capabilities of neuro-intensive care and neurological rehabilitation units. Nevertheless, indirect funduscopy remains the gold standard in the diagnosis of IOH, and should be performed in all patients suffering from SAH with a suspicious OUS as soon as the patient leaves life-supporting treatment.

## Conclusions

Ocular ultrasound is determined to be a valid bedside imaging technique with an accelerating learning curve in a short time period, facilitating an early diagnosis of intraocular hemorrhage and thus serving as a reliable screening tool for neuro-intensive care and neurological rehabilitation patients and for determining treatment decisions. Nevertheless funduscopy remains the gold standard of intraocular hemorrhages.

## References

[1] Weingeist TA, Goldman EJ, Folk JC, Packer AJ, Ossoinig KC (1986) Terson's Syndrome. Clinicopathologic correlations. Ophthalmology 93:1435–1442. 3808605 10.1016/s0161-6420(86)33548-6

[2] Litten M (1881) Ueber Einige von Allgemein-Klinischen Standpunkt aus Interessante Augenveränderungen. Berl Klin Wochenschr 18:23–27.

[3] Terson A (1900) De l'hemorrhagie dans le corps vitre au cours de l'hémorrhagie cérébrale. Clin Ophthalmol 6:309–312.

[4] Skevas C, Czorlich P, Knospe V, Stemplewitz B, Richard G, et al (2014) Terson's syndrome - Rate and surgical approach in patients with subarachnoid haemorrhage – A prospective interdisciplinary study. Ophthalmol 121:1628–1633. 10.1016/j.ophtha.2014.02.015 24697912

[5] Fountas KN, Kapsalaki EZ, Lee GP, Machinis TG, Grigorian AA, et al (2008) Terson haemorrhage in patients suffering aneurysmal subarachnoid haemorrhage: predisposing factors and prognostic significance. J Neurosurg 109:439–444. 18759574 10.3171/JNS/2008/109/9/0439

[6] Medele RJ, Stummer W, Mueller AJ, Steiger HJ, Reulen HJ (1998) Terson's syndrome in subarachnoid haemorrhage and servere brain injury accompanied by acutely raised intracranial pressure. J Neurosurg 88:851–854. 9576253 10.3171/jns.1998.88.5.0851

[7] Wietholter S, Steube D, Stolz HP (1998) Terson's syndrome: widespread ignored ophtalmologic complication of subarachnoid haemorrhage. Zentralbl. Neurochir 59:166–170. 9816667

[8] Garfinkle AM, Danys IR, Nicolle DA, Colohan AR, Brem S (1992) Terson's syndrome: a reversible cause of blindness following subarachnoid haemorrhage. J Neurosurg 76:766–771. 1564539 10.3171/jns.1992.76.5.0766

[9] Kuhn F, Morris R, Witherspoon CD, Mester V (1998) Terson syndrome. Results of vitrectomy and the significance of vitreous haemorrhage in patients with subarachnoid haemorrhage. Ophthalmol 105:472–477. 10.1016/S0161-6420(98)93030-5 9499778

[10] Lovelock CE, Rinkel GJ, Rothwell PM (2010) Time trends in outcome of subarachnoid hemorrhage: Population-based study and systematic review. Neurology 74:1494–1501. 20375310 10.1212/WNL.0b013e3181dd42b3 PMC2875923

[11] Frizzel RT, Kuhn F, Morris R, Quinn C, Fisher WS 3rd (1997) Screening for ocular haemorrhages in patients with ruptured cerebral aneuryms: a prospective study of 99 patients. Neurosurgery 41:529–533. 9310968 10.1097/00006123-199709000-00004

[12] Ropper AH (1986) Lateral displacement of the brain and level of consciousness in patients with an acute hemispheral mass. N Engl J Med 314:953–958. 3960059 10.1056/NEJM198604103141504

[13] Fisher CM (1967) Some Neuro-ophthalmological observations. J Neurol Neurosurg Psychiatry 30:383–392. 6062990 10.1136/jnnp.30.5.383 PMC496213

[14] Blaivas M, Theodoro D, Siezenski P (2003) Elevated intracranial pressure detected by bedside emergency ultrasonography of the optic nerve sheath. Acad Emer Med 10:376–381. 10.1111/j.1553-2712.2003.tb01352.x 12670853

[15] Ertl M, Barinka F, Torka E, Altmann M, Pfister K, et al (2014) Ocular Color-Coded Sonography – A Promising Tool for Neurologists and Intensive Care Physicians. Ultraschall in Med 35:422–431. 24647767 10.1055/s-0034-1366113

[16] Koskela E, Pekkola J, Kivisaari R, Kivelä T, Hernesniemi J, et al (2014) Comparison of CT and clinical findings of Terson's syndrome in 121 patients: a 1-year prospective study. J Neurosurg 120:1172–1178. 24628616 10.3171/2014.2.JNS131248

[17] Gauntt CD, Sherry RG, Kannan C (2007) Terson syndrome with bilateral optic nerve sheath haemorrhage. J Neuroophthalmol 27:193–194. 17895820 10.1097/WNO.0b013e31814b22dc

[18] Silverman RH, Lizzi FL, Ursea BG, Cozzarelli L, Ketterling JA, et al (2001) Safety levels for exposure of cornea and lens to very high-frequency ultrasound. J Ultrasound Med 20:979–986. 11549159 10.7863/jum.2001.20.9.979

[19] Torloni MR, Vedmedovska N, Merialdi M, Betrán AP, Allen T, et al (2009) ISUOG-WHO Fetal Growth Study Group. Safety of ultrasonography in pregnancy: WHO systematic review of the literature and meta-analysis. Ultrasound Obstet Gynecol 33:599–608. 19291813 10.1002/uog.6328

[20] Bello SO & Ekele BA (2012) On the safety of diagnostic ultrasound in pregnancy: Have we handled the available data correctly? Ann Afr Med 11:1–4. 22199039 10.4103/1596-3519.91006

[21] Rosa M (2012) US of the eye made easy. Radio Graphics 32:9–10.

[22] Berrocal T (1996) US and the color Doppler imaging of ocular and orbital disease. Radio Graphics 16:251–272. 10.1148/radiographics.16.2.8966285 8966285

[23] Leo M & Carmody K (2011) Sonography assessment of acute ocular pathology. Ultrasound Clin 6:227–234.

[24] Parchand S., Singh R, Bhalekar S (2014) Reliability of ocular ultrasonography findings for pre-surgical evaluation in various vitreo-retinal disorders. Semin Ophthalmol 29 236–241. 24409949 10.3109/08820538.2013.821506

[25] Yoonessi R, Hussain A, Jang T (2010) Bedside ocular ultrasound for the detection of retinal detachment in the emergency department. Acad Emerg Med 17:913–917. 20836770 10.1111/j.1553-2712.2010.00809.x

[26] Sharma T, Gopal L, Biswas J, Shanmugam MP, Bhende PS, et al (2002) Results of vitrectomy in Terson syndrome. Ophthalmic Surg Lasers 33:195–199. 12027098

[27] Czorlich P, Skevas C, Knospe V, Vettorazzi E, Richard G, et al. (2014) Terson syndrome in subarachnoid hemorrhage, intracerebral hemorrhage, and traumatic brain injury. Neurosurg Rev. E-pub ahead of print. 2014 Aug 31. doi: 10.1007/s10143-014-0564-4. 10.1007/s10143-014-0564-4 25173620

[28] Rubowitz A, Desai U (2006) Nontraumatic macular holes associated with Terson syndrome. Retina 26:230–232. 16467686 10.1097/00006982-200602000-00022

